# 1,4-Bis(1*H*-benzimidazol-1-yl)benzene

**DOI:** 10.1107/S1600536811031278

**Published:** 2011-08-11

**Authors:** Guo-Feng Sun, Jian-Ping Hu, Dian-Yong Tang, Yuan-Qin Zhang

**Affiliations:** a Molecular Design Center, College of Chemistry and Life Science, Leshan Normal University, Leshan 614000, Sichuan Province, People’s Republic of China

## Abstract

In the title compound, C_20_H_14_N_4_, the dihedral angles between the central benzene ring and the pendant benzimidazole ring systems are 46.60 (15) and 47.89 (16)°. The dihedral angle between the benzimidazole ring systems is 85.62 (12)° and the N atoms lie to the same side of the mol­ecule. In the crystal, mol­ecules are linked by C—H⋯N inter­actions and weak aromatic π–π stacking [shortest centroid–centroid separation = 3.770 (2) Å] is observed.

## Related literature

For background to benzimidazole derivatives as ligands in crystal engineering, see: Li *et al.* (2009 ▶); Vijayan *et al.* (2006 ▶).
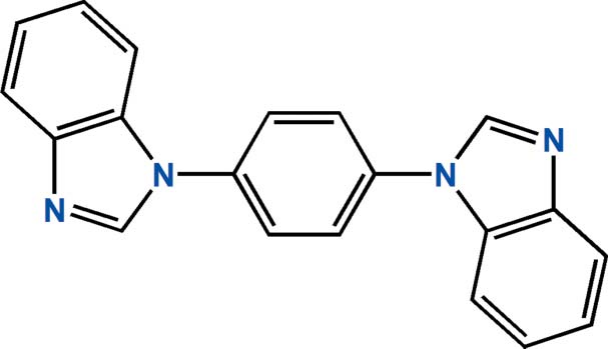


## Experimental

### 

#### Crystal data

C_20_H_14_N_4_
*M* *_r_* = 310.35
Orthorhombic, 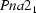

*a* = 9.5458 (19) Å
*b* = 20.499 (4) Å
*c* = 7.9283 (16) Å
*V* = 1551.4 (5) Å^3^
*Z* = 4
Mo *K*α radiation
μ = 0.08 mm^−1^
*T* = 293 K
0.25 × 0.22 × 0.18 mm

#### Data collection

Rigaku Mercury CCD diffractometer
Absorption correction: multi-scan (*CrystalClear*; Rigaku/MSC, 2005 ▶) *T*_min_ = 0.980, *T*_max_ = 0.985
12744 measured reflections
1479 independent reflections
1298 reflections with *I* > 2σ(*I*)
*R*_int_ = 0.075

#### Refinement

*R*[*F* ^2^ > 2σ(*F* ^2^)] = 0.051
*wR*(*F* ^2^) = 0.104
*S* = 1.17
1479 reflections
231 parameters
1 restraint
H-atom parameters constrained
Δρ_max_ = 0.14 e Å^−3^
Δρ_min_ = −0.16 e Å^−3^

### 

Data collection: *CrystalClear* (Rigaku/MSC, 2005 ▶); cell refinement: *CrystalClear*; data reduction: *CrystalClear*; program(s) used to solve structure: *SHELXS97* (Sheldrick, 2008 ▶); program(s) used to refine structure: *SHELXL97* (Sheldrick, 2008 ▶); molecular graphics: *SHELXTL* (Sheldrick, 2008 ▶); software used to prepare material for publication: *SHELXTL* (Sheldrick, 2008 ▶).

## Supplementary Material

Crystal structure: contains datablock(s) I, global. DOI: 10.1107/S1600536811031278/hb6340sup1.cif

Structure factors: contains datablock(s) I. DOI: 10.1107/S1600536811031278/hb6340Isup2.hkl

Supplementary material file. DOI: 10.1107/S1600536811031278/hb6340Isup3.cml

Additional supplementary materials:  crystallographic information; 3D view; checkCIF report

## Figures and Tables

**Table 1:** Hydrogen-bond geometry (Å, °)

*D*—H⋯*A*	*D*—H	H⋯*A*	*D*⋯*A*	*D*—H⋯*A*
C1—H1⋯N4^i^	0.93	2.50	3.359 (5)	153
C6—H6⋯N4^ii^	0.93	2.56	3.447 (5)	159

Symmetry codes: (i) 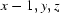; (ii) 
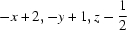
.

**Fig. 1.**
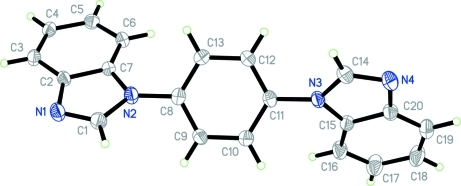
The molecular structure of (I). Displacement ellipsoids are drawn at the 30% probability level and H atoms are shown as small spheres of arbitrary radius.

**Fig. 2.**
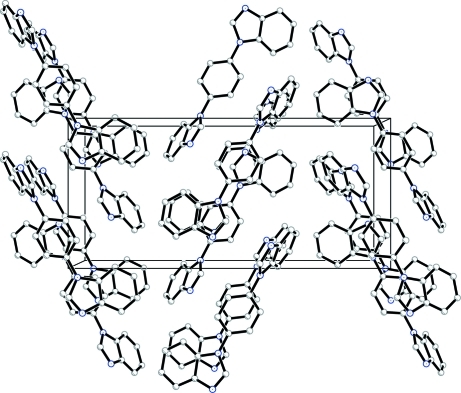
The crystal packing for (I).

## supplementary crystallographic information

### Comment

In recent years, benzimidazole has been well used in crystal engineering, because they exhibit a strong networking ability (Vijayan *et al.*, 2006). To our knowledge, the research on benzimidazole ligands bearing rigid spacers is still less developed (Li *et al.*, 2009), and the title compound was well designed for building polymer architecture. We report here the structure and conformation of a rigid benzimidazole derivative, and further explore the ligand coordination. As shown in Fig. 1, the title compound is *trans*-conformation and tends to *trans*-coordination. The molecule has no inversion centre because two benzimidazole rings are not coplanar.

### Experimental

The ligand 1,4-di(1*H*-benzimidazol-1-yl)benzene was prepared by a modified method (Li *et al.*, 2009). A mixture of 1,4-dibromophenyl (3.72 g, 12.0 mmol), benzimidazole (4.25 g, 36.0 mmol), CuI (0.38 g, 2.0 mmol), 1,10-phenanthroline (0.72 g, 4.0 mmol), and Cs_2_CO_3_ (2.48 g, 18.0 mmol) was suspended in DMF (50 ml) and refluxed for 48 h to afford (I) as off-white powder, yield: 25% (based on 1,4-dibromophenyl), Mp: 291°C. Colourless blocks of (I) were obtained by recrystallizing from a mixed solvent of methanol and water (1:1).

### Refinement

C-bound H atoms were positioned geometrically and refined in the riding-model approximation, with C—H = 0.93Å and *U*_iso_(H) = 1.2*U*_eq_. Anomalous dispersion was negligible and Friedel pairs were merged before refinement.

### Crystal data

**Table:**

C_20_H_14_N_4_	*D*_x_ = 1.329 Mg m^−^^3^
*M**_r_* = 310.35	Melting point: 564 K
Orthorhombic, *P**n**a*2_1_	Mo *K*α radiation, λ = 0.71073 Å
Hall symbol: P 2c -2n	Cell parameters from 13068 reflections
*a* = 9.5458 (19) Å	θ = 3.3–27.6°
*b* = 20.499 (4) Å	µ = 0.08 mm^−^^1^
*c* = 7.9283 (16) Å	*T* = 293 K
*V* = 1551.4 (5) Å^3^	Block, colorless
*Z* = 4	0.25 × 0.22 × 0.18 mm
*F*(000) = 648	

### Data collection

**Table:**

Rigaku Mercury CCD diffractometer	1479 independent reflections
Radiation source: fine-focus sealed tube	1298 reflections with *I* > 2σ(*I*)
graphite	*R*_int_ = 0.075
Detector resolution: 9 pixels mm^-1^	θ_max_ = 25.0°, θ_min_ = 3.5°
ω scans	*h* = −11→11
Absorption correction: multi-scan (*CrystalClear*; Rigaku/MSC, 2005)	*k* = −24→24
*T*_min_ = 0.980, *T*_max_ = 0.985	*l* = −9→9
12744 measured reflections	

### Refinement

**Table:**

Refinement on *F*^2^	Primary atom site location: structure-invariant direct methods
Least-squares matrix: full	Secondary atom site location: difference Fourier map
*R*[*F*^2^ > 2σ(*F*^2^)] = 0.051	Hydrogen site location: inferred from neighbouring sites
*wR*(*F*^2^) = 0.104	H-atom parameters constrained
*S* = 1.17	*w* = 1/[σ^2^(*F*_o_^2^) + (0.0488*P*)^2^ + 0.1271*P*] where *P* = (*F*_o_^2^ + 2*F*_c_^2^)/3
1479 reflections	(Δ/σ)_max_ < 0.001
231 parameters	Δρ_max_ = 0.14 e Å^−^^3^
1 restraint	Δρ_min_ = −0.16 e Å^−^^3^
0 constraints	

### Special details

**Table:**

Geometry. All e.s.d.'s (except the e.s.d. in the dihedral angle between two l.s. planes) are estimated using the full covariance matrix. The cell e.s.d.'s are taken into account individually in the estimation of e.s.d.'s in distances, angles and torsion angles; correlations between e.s.d.'s in cell parameters are only used when they are defined by crystal symmetry. An approximate (isotropic) treatment of cell e.s.d.'s is used for estimating e.s.d.'s involving l.s. planes.
Refinement. Refinement of *F*^2^ against ALL reflections. The weighted *R*-factor *wR* and goodness of fit *S* are based on *F*^2^, conventional *R*-factors *R* are based on *F*, with *F* set to zero for negative *F*^2^. The threshold expression of *F*^2^ > σ(*F*^2^) is used only for calculating *R*-factors(gt) *etc*. and is not relevant to the choice of reflections for refinement. *R*-factors based on *F*^2^ are statistically about twice as large as those based on *F*, and *R*- factors based on ALL data will be even larger.

### Fractional atomic coordinates and isotropic or equivalent isotropic displacement parameters (Å^2^)

**Table:**

	*x*	*y*	*z*	*U*_iso_*/*U*_eq_	
N1	0.2140 (3)	0.55131 (15)	0.6320 (4)	0.0462 (8)	
N2	0.4461 (3)	0.53457 (14)	0.6010 (4)	0.0411 (8)	
N3	0.9643 (3)	0.40659 (14)	0.6681 (4)	0.0383 (7)	
N4	1.1632 (3)	0.37523 (15)	0.7980 (4)	0.0479 (9)	
C1	0.3178 (4)	0.51125 (18)	0.6527 (5)	0.0452 (9)	
H1	0.3065	0.4699	0.6993	0.043 (10)*	
C2	0.2750 (3)	0.60700 (16)	0.5602 (4)	0.0361 (8)	
C3	0.2141 (4)	0.66544 (17)	0.5108 (5)	0.0427 (9)	
H3	0.1189	0.6729	0.5263	0.036 (9)*	
C4	0.2986 (4)	0.71179 (17)	0.4385 (5)	0.0421 (9)	
H4	0.2598	0.7513	0.4047	0.063 (13)*	
C5	0.4412 (4)	0.70086 (17)	0.4146 (5)	0.0442 (10)	
H5	0.4948	0.7331	0.3629	0.046 (11)*	
C6	0.5059 (4)	0.64388 (17)	0.4649 (5)	0.0431 (9)	
H6	0.6014	0.6371	0.4499	0.045 (10)*	
C7	0.4197 (3)	0.59693 (16)	0.5399 (5)	0.0364 (8)	
C8	0.5788 (3)	0.50222 (17)	0.6151 (4)	0.0379 (9)	
C9	0.5905 (4)	0.43768 (17)	0.5667 (5)	0.0419 (9)	
H9	0.5134	0.4158	0.5226	0.058 (12)*	
C10	0.7177 (3)	0.40549 (17)	0.5842 (5)	0.0408 (9)	
H10	0.7256	0.3618	0.5539	0.037 (9)*	
C11	0.8327 (3)	0.43899 (17)	0.6469 (5)	0.0366 (8)	
C12	0.8204 (4)	0.50358 (17)	0.6927 (5)	0.0421 (9)	
H12	0.8982	0.5260	0.7330	0.047 (11)*	
C13	0.6930 (4)	0.53532 (18)	0.6794 (5)	0.0455 (10)	
H13	0.6843	0.5786	0.7130	0.037 (10)*	
C14	1.0470 (4)	0.40901 (19)	0.8087 (5)	0.0443 (9)	
H14	1.0229	0.4329	0.9042	0.046 (11)*	
C15	1.0353 (3)	0.36720 (16)	0.5527 (5)	0.0391 (9)	
C16	1.0063 (4)	0.3484 (2)	0.3891 (6)	0.0523 (11)	
H16	0.9241	0.3610	0.3355	0.064 (13)*	
C17	1.1043 (4)	0.3103 (2)	0.3088 (6)	0.0662 (12)	
H17	1.0880	0.2971	0.1983	0.086 (16)*	
C18	1.2279 (5)	0.2907 (2)	0.3899 (6)	0.0650 (13)	
H18	1.2914	0.2644	0.3325	0.055 (12)*	
C19	1.2571 (4)	0.30983 (19)	0.5533 (6)	0.0525 (10)	
H19	1.3397	0.2973	0.6065	0.065 (13)*	
C20	1.1584 (4)	0.34847 (17)	0.6357 (5)	0.0423 (9)	

### Atomic displacement parameters (Å^2^)

**Table:**

	*U*^11^	*U*^22^	*U*^33^	*U*^12^	*U*^13^	*U*^23^
N1	0.0327 (17)	0.0505 (18)	0.056 (2)	0.0003 (15)	0.0048 (16)	0.0058 (17)
N2	0.0318 (16)	0.0426 (18)	0.049 (2)	0.0027 (13)	0.0035 (15)	0.0057 (15)
N3	0.0292 (15)	0.0410 (16)	0.0448 (18)	0.0019 (13)	−0.0052 (14)	0.0046 (16)
N4	0.040 (2)	0.058 (2)	0.046 (2)	0.0025 (16)	−0.0089 (16)	0.0052 (18)
C1	0.041 (2)	0.038 (2)	0.057 (2)	−0.0039 (17)	0.010 (2)	0.009 (2)
C2	0.0285 (17)	0.0417 (19)	0.038 (2)	−0.0029 (16)	0.0033 (17)	−0.0012 (17)
C3	0.034 (2)	0.047 (2)	0.047 (2)	0.0072 (17)	0.0010 (17)	−0.0050 (19)
C4	0.039 (2)	0.0353 (18)	0.052 (2)	0.0059 (17)	0.0013 (19)	0.0000 (19)
C5	0.044 (2)	0.038 (2)	0.051 (2)	−0.0019 (18)	0.0041 (19)	0.003 (2)
C6	0.031 (2)	0.047 (2)	0.052 (2)	−0.0003 (17)	0.0011 (19)	−0.0041 (19)
C7	0.0305 (18)	0.0354 (19)	0.043 (2)	0.0001 (15)	0.0014 (17)	−0.0035 (19)
C8	0.0363 (19)	0.041 (2)	0.036 (2)	0.0057 (15)	0.0021 (16)	0.0033 (17)
C9	0.0316 (19)	0.042 (2)	0.052 (2)	−0.0021 (16)	−0.0049 (18)	0.0017 (19)
C10	0.039 (2)	0.038 (2)	0.046 (2)	0.0017 (17)	−0.0042 (17)	−0.0015 (18)
C11	0.0318 (19)	0.044 (2)	0.0339 (19)	0.0024 (15)	−0.0022 (17)	0.0042 (18)
C12	0.034 (2)	0.043 (2)	0.049 (2)	−0.0014 (17)	−0.0066 (17)	−0.0030 (19)
C13	0.044 (2)	0.039 (2)	0.054 (3)	0.0039 (17)	−0.0021 (19)	−0.0105 (19)
C14	0.039 (2)	0.053 (2)	0.041 (2)	−0.0013 (19)	−0.0063 (19)	0.001 (2)
C15	0.0338 (19)	0.0346 (18)	0.049 (2)	0.0030 (15)	−0.0013 (18)	0.0040 (18)
C16	0.048 (2)	0.061 (3)	0.048 (2)	0.013 (2)	−0.013 (2)	−0.007 (2)
C17	0.064 (3)	0.079 (3)	0.056 (3)	0.024 (2)	−0.004 (2)	−0.015 (3)
C18	0.057 (3)	0.068 (3)	0.070 (3)	0.024 (2)	0.003 (2)	−0.010 (3)
C19	0.042 (2)	0.056 (2)	0.060 (3)	0.011 (2)	−0.005 (2)	0.006 (2)
C20	0.037 (2)	0.041 (2)	0.049 (2)	0.0012 (16)	−0.0034 (19)	0.005 (2)

### Geometric parameters (Å, °)

**Table:**

N1—C1	1.298 (5)	C8—C13	1.381 (5)
N1—C2	1.402 (4)	C8—C9	1.382 (5)
N2—C1	1.377 (4)	C9—C10	1.389 (5)
N2—C7	1.390 (4)	C9—H9	0.9300
N2—C8	1.434 (4)	C10—C11	1.387 (5)
N3—C14	1.367 (4)	C10—H10	0.9301
N3—C15	1.396 (5)	C11—C12	1.378 (5)
N3—C11	1.431 (4)	C12—C13	1.384 (5)
N4—C14	1.310 (4)	C12—H12	0.9299
N4—C20	1.400 (5)	C13—H13	0.9301
C1—H1	0.9301	C14—H14	0.9299
C2—C3	1.388 (5)	C15—C16	1.381 (6)
C2—C7	1.406 (4)	C15—C20	1.400 (5)
C3—C4	1.372 (5)	C16—C17	1.374 (6)
C3—H3	0.9300	C16—H16	0.9299
C4—C5	1.393 (5)	C17—C18	1.402 (6)
C4—H4	0.9299	C17—H17	0.9300
C5—C6	1.380 (5)	C18—C19	1.382 (6)
C5—H5	0.9301	C18—H18	0.9300
C6—C7	1.399 (5)	C19—C20	1.394 (5)
C6—H6	0.9300	C19—H19	0.9301
			
C1—N1—C2	104.4 (3)	C10—C9—H9	120.0
C1—N2—C7	105.2 (3)	C11—C10—C9	119.5 (3)
C1—N2—C8	127.0 (3)	C11—C10—H10	120.3
C7—N2—C8	127.8 (3)	C9—C10—H10	120.2
C14—N3—C15	106.0 (3)	C12—C11—C10	120.2 (3)
C14—N3—C11	125.8 (3)	C12—C11—N3	119.3 (3)
C15—N3—C11	128.2 (3)	C10—C11—N3	120.5 (3)
C14—N4—C20	103.8 (3)	C11—C12—C13	120.4 (3)
N1—C1—N2	115.0 (3)	C11—C12—H12	119.8
N1—C1—H1	122.5	C13—C12—H12	119.8
N2—C1—H1	122.5	C8—C13—C12	119.4 (3)
C3—C2—N1	130.0 (3)	C8—C13—H13	120.3
C3—C2—C7	120.4 (3)	C12—C13—H13	120.3
N1—C2—C7	109.6 (3)	N4—C14—N3	114.6 (4)
C4—C3—C2	118.0 (3)	N4—C14—H14	122.7
C4—C3—H3	121.0	N3—C14—H14	122.7
C2—C3—H3	120.9	C16—C15—N3	132.8 (3)
C3—C4—C5	121.3 (3)	C16—C15—C20	122.2 (4)
C3—C4—H4	119.3	N3—C15—C20	104.9 (3)
C5—C4—H4	119.4	C17—C16—C15	117.2 (4)
C6—C5—C4	122.3 (4)	C17—C16—H16	121.4
C6—C5—H5	118.9	C15—C16—H16	121.4
C4—C5—H5	118.9	C16—C17—C18	121.5 (5)
C5—C6—C7	116.2 (3)	C16—C17—H17	119.2
C5—C6—H6	121.9	C18—C17—H17	119.3
C7—C6—H6	121.9	C19—C18—C17	121.2 (4)
N2—C7—C6	132.4 (3)	C19—C18—H18	119.4
N2—C7—C2	105.9 (3)	C17—C18—H18	119.4
C6—C7—C2	121.7 (3)	C18—C19—C20	117.7 (4)
C13—C8—C9	120.6 (3)	C18—C19—H19	121.2
C13—C8—N2	119.9 (3)	C20—C19—H19	121.1
C9—C8—N2	119.5 (3)	C19—C20—N4	129.2 (3)
C8—C9—C10	119.8 (3)	C19—C20—C15	120.2 (4)
C8—C9—H9	120.1	N4—C20—C15	110.6 (3)
			
C2—N1—C1—N2	−0.1 (4)	C14—N3—C11—C12	46.4 (5)
C7—N2—C1—N1	−0.1 (5)	C15—N3—C11—C12	−133.0 (4)
C8—N2—C1—N1	177.2 (4)	C14—N3—C11—C10	−132.3 (4)
C1—N1—C2—C3	179.9 (4)	C15—N3—C11—C10	48.3 (5)
C1—N1—C2—C7	0.2 (4)	C10—C11—C12—C13	1.1 (6)
N1—C2—C3—C4	−178.1 (4)	N3—C11—C12—C13	−177.6 (4)
C7—C2—C3—C4	1.5 (5)	C9—C8—C13—C12	0.9 (6)
C2—C3—C4—C5	0.2 (6)	N2—C8—C13—C12	179.9 (3)
C3—C4—C5—C6	−1.4 (6)	C11—C12—C13—C8	−1.8 (6)
C4—C5—C6—C7	0.8 (6)	C20—N4—C14—N3	0.6 (4)
C1—N2—C7—C6	−177.3 (4)	C15—N3—C14—N4	−0.5 (4)
C8—N2—C7—C6	5.4 (7)	C11—N3—C14—N4	−179.9 (3)
C1—N2—C7—C2	0.2 (4)	C14—N3—C15—C16	−178.0 (4)
C8—N2—C7—C2	−177.1 (3)	C11—N3—C15—C16	1.4 (6)
C5—C6—C7—N2	178.1 (4)	C14—N3—C15—C20	0.1 (4)
C5—C6—C7—C2	1.0 (6)	C11—N3—C15—C20	179.6 (3)
C3—C2—C7—N2	180.0 (3)	N3—C15—C16—C17	177.6 (4)
N1—C2—C7—N2	−0.3 (4)	C20—C15—C16—C17	−0.3 (6)
C3—C2—C7—C6	−2.2 (6)	C15—C16—C17—C18	0.6 (7)
N1—C2—C7—C6	177.6 (4)	C16—C17—C18—C19	−1.0 (7)
C1—N2—C8—C13	−131.9 (4)	C17—C18—C19—C20	1.0 (6)
C7—N2—C8—C13	44.8 (5)	C18—C19—C20—N4	−178.6 (4)
C1—N2—C8—C9	47.1 (5)	C18—C19—C20—C15	−0.6 (5)
C7—N2—C8—C9	−136.2 (4)	C14—N4—C20—C19	177.6 (4)
C13—C8—C9—C10	0.7 (6)	C14—N4—C20—C15	−0.5 (4)
N2—C8—C9—C10	−178.3 (3)	C16—C15—C20—C19	0.3 (5)
C8—C9—C10—C11	−1.4 (6)	N3—C15—C20—C19	−178.1 (3)
C9—C10—C11—C12	0.5 (5)	C16—C15—C20—N4	178.6 (4)
C9—C10—C11—N3	179.2 (3)	N3—C15—C20—N4	0.3 (4)

### Hydrogen-bond geometry (Å, °)

**Table:**

*D*—H···*A*	*D*—H	H···*A*	*D*···*A*	*D*—H···*A*
C1—H1···N4^i^	0.93	2.50	3.359 (5)	153
C6—H6···N4^ii^	0.93	2.56	3.447 (5)	159

Symmetry codes: (i) *x*−1, *y*, *z*; (ii) −*x*+2, −*y*+1, *z*−1/2.
